# AoI-Aware Data Collection in Heterogeneous UAV-Assisted WSNs: Strong-Agent Coordinated Coverage and Vicsek-Driven Weak-Swarm Control

**DOI:** 10.3390/s26020419

**Published:** 2026-01-08

**Authors:** Lin Huang, Lanhua Li, Songhan Zhao, Daiming Qu, Jing Xu

**Affiliations:** 1 School of Electronic Information and Communications, Huazhong University of Science and Technology, Wuhan 430074, China; d201980615@hust.edu.cn (L.H.); qudaiming@hust.edu.cn (D.Q.); 2 Wuhan Maritime Communication Research Institute, Wuhan 430079, China; 3 School of Intelligent Systems Engineering, Shenzhen Campus of Sun Yat-Sen University, Shenzhen 518107, China; lilh65@mail.sysu.edu.cn (L.L.); zhaosh55@mail2.sysu.edu.cn (S.Z.)

**Keywords:** heterogeneous UAV system, decentralized swarm control, Voronoi partition, Vicsek model, AoI-aware data collection

## Abstract

Unmanned aerial vehicle (UAV) swarms offer an efficient solution for data collection from widely distributed ground users (GUs). However, incomplete environment information and frequent changes make it challenging for standard centralized planning or pure reinforcement learning approaches to simultaneously maintain global solution quality and local flexibility. We propose a hierarchical data collection framework for heterogeneous UAV-assisted wireless sensor networks (WSNs). A small set of high-capability UAVs (H-UAVs), equipped with substantial computational and communication resources, coordinate regional coverage, trajectory planning, and uplink transmission control for numerous resource-constrained low-capability UAVs (L-UAVs) across power-Voronoi-partitioned areas using multi-agent deep reinforcement learning (MADRL). Specifically, we employ Multi-Agent Deep Deterministic Policy Gradient (MADDPG) to enhance H-UAVs’ decision-making capabilities and enable coordinated actions. The partitions are dynamically updated based on GUs’ data generation rates and L-UAV density to balance workload and adapt to environmental dynamics. Concurrently, a large number of L-UAVs with limited onboard resources perform self-organized data collection from GUs and execute opportunistic relaying to a remote access point (RAP) via H-UAVs. Within each Voronoi cell, L-UAV motion follows a weighted Vicsek model that incorporates GUs’ age of information (AoI), link quality, and congestion avoidance. This spatial decomposition combined with decentralized weak-swarm control enables scalability to large-scale L-UAV deployments. Experiments demonstrate that the proposed strong and weak agent MADDPG (SW-MADDPG) scheme reduces AoI by 30% and 21% compared to No-Voronoi and Heuristic-HUAV baselines, respectively.

## 1. Introduction

### 1.1. Motivations and Challenges

The proliferation of Internet of Things (IoT) devices and wireless sensor networks (WSNs) has created an unprecedented demand for efficient data collection from geographically dispersed ground users (GUs). Traditional infrastructure-based solutions face significant limitations in coverage, deployment costs, and adaptability to dynamic environments. Unmanned aerial vehicle (UAV) swarms have emerged as a promising alternative, offering flexible, cost-effective, and rapidly deployable platforms for aerial data collection and relaying in scenarios such as disaster response, environmental monitoring, precision agriculture, and smart cities.

Despite their potential, UAV swarm-assisted data collection faces several critical challenges. First, the scalability-coordination dilemma arises when deploying large-scale UAV swarms: centralized control schemes suffer from prohibitive computational complexity and communication overhead, while fully decentralized approaches struggle to achieve globally optimal performance. Second, environmental uncertainty and dynamics pose significant obstacles. The incomplete knowledge of GU distributions, time-varying data generation rates, and unpredictable channel conditions makes it difficult for traditional model-based planning methods to maintain robustness. Third, resource heterogeneity among UAVs complicates system design. While high-capability UAVs can handle complex coordination tasks, resource-constrained UAVs require lightweight, self-organized control mechanisms. Fourth, ensuring timely data delivery while managing the age of information (AoI) across distributed GUs demands intelligent trajectory planning and transmission scheduling that balances exploration, exploitation, and energy efficiency.

Existing approaches typically fall into two categories: optimization-based methods that require perfect environmental knowledge and cannot adapt to real-time changes, or pure learning-based methods that lack global coordination and struggle with sample efficiency in multi-agent settings. These limitations motivate the need for a hierarchical framework that combines global learning-based coordination with local self-organized control, enabling both scalability and adaptation in heterogeneous UAV swarm-assisted data collection.

### 1.2. Solutions and Contributions

To address the aforementioned challenges, we propose a novel hierarchical data collection framework that leverages the complementary strengths of heterogeneous UAVs through spatial decomposition and multi-level decision-making. Our approach integrates multi-agent deep reinforcement learning (MADRL) for high-level coordination with bio-inspired swarm intelligence for low-level execution.

The main contributions of this paper are outlined as follows:

- Hierarchical heterogeneous UAV architecture: We design a two-tier framework where a small number of high-capability H-UAVs learn to coordinate regional coverage and manage large swarms of resource-constrained L-UAVs. This architecture naturally decomposes the complex global optimization problem into manageable subproblems while maintaining coordination through power–Voronoi partitioning that adapts to workload dynamics and UAV density.
- MADRL-based intelligent coordination: We formulate the H-UAV coordination problem as a partially observable Markov decision process (POMDP) and employ multi-agent deep deterministic policy gradient (MADDPG) with centralized training and decentralized execution. This enables H-UAVs to learn coordinated policies for trajectory planning, partition management, and uplink transmission control without requiring complete environmental models, while adapting to time-varying GU demands and channel conditions.
- Scalable self-organized L-UAV swarm control: We develop a weighted Vicsek model that incorporates task-specific factors, including the GUs’ AoI, wireless link quality, and congestion avoidance, to guide L-UAV motion within Voronoi cells. This decentralized mechanism requires only local information exchange, enabling efficient scaling to large-scale L-UAVs while achieving emergent collective behaviors such as coverage maximization and load balancing.

The remainder of this paper is organized as follows: Section 2 reviews related work. Section 3 presents the system model and problem formulation. Section 4 details the proposed hierarchical framework and describes the MADRL algorithm for H-UAVs and the weighted Vicsek model for L-UAVs. Section 5 presents simulation results and performance analysis. Section 6 discusses the advantages and limitations of the proposed method. Finally, Section 7 concludes the paper and outlines future research directions.

## 2. Related Work

### 2.1. UAV-Assisted Data Collection in Wireless Sensor Networks

UAV-assisted data collection has been extensively studied as a flexible and cost-effective solution for WSN/IoT monitoring. Early efforts largely considered single-UAV settings, where the core problem is to design an energy- and time-efficient tour to visit (or communicate with) distributed sensors. Typical formulations include TSP-like trajectory design with communication range constraints to reduce the search space and improve energy efficiency [1], and hover-and-collect paradigms with clustering/cluster-head selection to enhance uplink reliability and collection throughput under energy budgets [2].

As the scope expands to multi-UAV systems, the literature has explored cooperative path planning and coordination mechanisms to improve coverage and reduce latency, including energy-aware routing and reliability-oriented data collection [3], joint task allocation and communication resource optimization [4], and integrated charging/scheduling in UAV-assisted edge/IoT networks [5]. More recently, researchers have explored age-of-information (AoI)-aware trajectory planning for UAVs. For instance, Ref. [6] proposed a deep learning approach for AoI-aware trajectory planning in intelligent transportation systems, while Ref. [7] investigated multi-UAV enabled age-optimal data collection in large-scale IoT systems, demonstrating that explicit AoI optimization can significantly improve information freshness compared to traditional latency- or throughput-centric designs.

However, these approaches typically assume limited and homogeneous UAV capabilities, static or fully observable environments where sensor locations and data generation patterns are known a priori, and performance metrics focused primarily on coverage rate, energy consumption, or average delay. Consequently, most solutions rely on centralized optimization frameworks, such as mixed-integer linear programming (MILP), dynamic programming, or heuristic algorithms, that do not scale well beyond a handful of UAVs. Moreover, they struggle to adapt to highly dynamic scenarios characterized by time-varying data demands, uncertain channel conditions, and partial observability. In contrast, our work addresses large-scale heterogeneous UAV swarms operating under incomplete information, requiring a fundamentally different approach that combines hierarchical learning with decentralized control.

### 2.2. Reinforcement Learning for UAV Control and Network Optimization

Reinforcement learning (RL) has become a prominent approach for UAV control and wireless-network optimization under uncertainty. In single-agent settings, deep RL methods, such as Deep Q-Networks (DQN) [8], Proximal Policy Optimization (PPO) [9], and Asynchronous Advantage Actor-Critic (A3C) [10], have been used for trajectory planning, obstacle avoidance, and resource control, benefiting from model-free learning and adaptability. In the context of AoI optimization, the authors proposed a reinforcement learning framework for optimizing AoI in RF-powered communication systems, demonstrating the potential of RL to handle stochastic arrivals and energy constraints [11].

For multi-UAV coordination, multi-agent RL (MARL) has drawn growing attention, with representative methods including value factorization (e.g., QMIX) [12], counterfactual credit assignment (e.g., COMA) [13], and actor–critic approaches for continuous control (e.g., MADDPG) [14]. The centralized training and decentralized execution (CTDE) paradigm [15] is particularly relevant to UAV networks, since it enables agents to learn coordinated strategies using global information during training while relying only on local observations at deployment time. Recent advances in MARL for AoI minimization include [16], which specifically addresses AoI minimization in UAV-aided networks using multi-agent reinforcement learning (MARL), demonstrating superior performance over traditional scheduling policies.

Furthermore, hierarchical MARL architectures have emerged as a promising direction for managing complexity in large-scale multi-agent systems. The authors in [17] introduced hierarchical deep multiagent reinforcement learning with temporal abstraction, enabling agents to learn at multiple time scales. In the UAV domain, hierarchical MARL is applied to multi-UAV-assisted mobile edge computing, showing that decomposing the problem into high-level task assignment and low-level trajectory control significantly improves scalability and convergence speed [18].

Despite these advances, existing MARL approaches face several challenges: limited sample efficiency in high-dimensional state-action spaces, scalability issues as the number of agents increases, difficulty handling partial observability and non-stationarity induced by concurrent learning. Critically, most MARL research assumes homogeneous agents with identical capabilities and focuses on scenarios involving several agents. In contrast, our framework explicitly models heterogeneous UAV capabilities through a hierarchical structure where high-capability H-UAVs learn coordinated policies while managing large swarms of resource-constrained L-UAVs.

### 2.3. Swarm Intelligence and Bio-Inspired Control

Bio-inspired swarm intelligence provides an alternative coordination paradigm characterized by local interactions, scalability, and robustness. Classic models such as the Vicsek flocking dynamics [19] and boid-style rules [20] illustrate how simple alignment/cohesion/separation mechanisms can produce emergent group behaviors. Particle swarm optimization (PSO) [21] has also been widely adopted as a distributed search heuristic for high-dimensional optimization problems and has been adapted for UAV-related planning tasks.

In UAV swarms, bio-inspired methods have been applied to formation and mission execution [22], communication coverage improvement through heterogeneous PSO variants [23], and cooperative multi-target tracking with collision avoidance [24]. Beyond motion coordination, lightweight mechanisms for large-scale UAV networking have been studied via consensus-style protocols [24] and hybrid global–local planning that integrates PSO with artificial potential fields for 3D path planning and obstacle avoidance [23].

For spatial coverage and resource partitioning, Voronoi-based methods have proven effective. The authors in [25] proposed decentralized autonomous navigation using Voronoi partitioning for air pollution sensing, while the authors in [26] introduced power-Voronoi diagrams for joint trajectory design, resource allocation, and task offloading in multi-UAV mobile edge computing, demonstrating that adaptive partitioning based on service capability can significantly improve load balancing and energy efficiency.

In terms of advanced multiple access techniques, NOMA has been integrated with UAV communications to enhance spectral efficiency. The authors in [27] investigated NOMA-aided UAV communications with joint trajectory optimization and power allocation for uplink transmission, showing substantial gains over orthogonal multiple access. Similarly, the authors in [28] explored trajectory design and power control for multi-UAV-assisted wireless networks with NOMA using machine learning, demonstrating improved throughput and fairness in dense deployment scenarios.

Although these decentralized approaches are attractive for large swarms due to their low communication and computation overhead, they are often reactive and may lack explicit mechanisms to optimize system-level objectives under complex constraints, such as AoI-aware scheduling, queue stability across multi-hop relaying, channel-aware association, and dynamic workload balancing. In particular, incorporating AoI-driven priorities and link-feasibility constraints into purely bio-inspired motion rules is nontrivial without a higher-level coordinator.

### 2.4. Summary and Positioning of This Work

In summary, prior studies in UAV-assisted data collection provide strong foundations in trajectory optimization and multi-UAV cooperation, MARL contributes adaptability under uncertainty, and swarm intelligence offers scalable decentralized control. However, a remaining challenge is to simultaneously achieve scalable coordination for large swarms, adaptability to time-varying traffic and channels under partial observability, explicit AoI-aware end-to-end control across multi-hop queues, and practical heterogeneity-aware designs that leverage a small number of resource-rich UAVs to guide many resource-limited agents.

Our work addresses these gaps by proposing a hierarchical framework that integrates MADDPG-based coordination for H-UAVs with AoI-aware Vicsek-driven decentralized control for L-UAV swarms, coupled through adaptive power–Voronoi partitioning and a two-stage communication protocol (GU→L-UAV and L-UAV→H-UAV→RAP). This design aims to retain the scalability and robustness of swarm control while enabling learning-based global decision-making for AoI minimization and workload balancing. The main notations and symbols used in this work are listed in Table 1.

## 3. System Model

**Figure 1:**
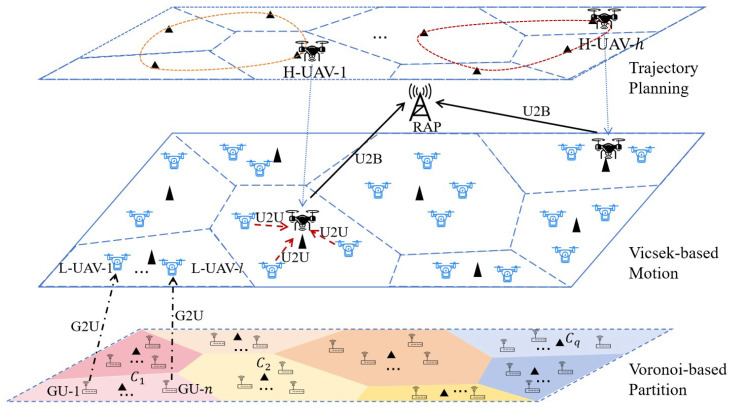
A heterogeneous UAV-assisted WSN.

As illustrated in Figure 1, we consider a heterogeneous UAV-assisted WSN comprising $N_{h}$ high-capability UAVs (H-UAVs), $N_{l}$ low-capability UAVs (L-UAVs), and $N_{g}$ spatially distributed ground users (GUs), denoted by the sets $N_{H}=\left\{1,2,\ldots ,N_{h}\right\}$, $N_{L}=\left\{1,2,\ldots ,N_{l}\right\}$, and $N_{G}=\left\{1,2,\ldots ,N_{g}\right\}$, respectively. The GUs are stationary nodes that generate status update packets at heterogeneous rates $\lambda_{i}$ for all $i\in N_{G}$, serving as sources of time-sensitive information. The operational area is denoted by $\Omega \subset R^{2}$.

A large swarm of resource-constrained L-UAVs is deployed to collect data from associated GUs and relay it opportunistically to H-UAVs. These L-UAVs operate within dynamically defined power-Voronoi cells, each governed by an H-UAV. Within their respective cells, L-UAVs perform self-organized mobility based on a weighted Vicsek model, enabling coordinated exploration and efficient data gathering while respecting spatial boundaries. In contrast, the H-UAVs serve as aerial coordinators in this hierarchical architecture, responsible for optimizing their trajectories to maintain connectivity and coverage, managing adaptive power-Voronoi partitioning through dynamically updated weights to balance workload and mitigate congestion, coordinating uplink data aggregation from L-UAVs using non-orthogonal multiple access (NOMA), and forwarding aggregated data to a remote access point (RAP) via orthogonal frequency-division multiple access (OFDMA) backhaul links. The RAP acts as the central data fusion center, receiving and processing status updates from all H-UAVs. This hierarchical, two-tier relaying structure, where L-UAVs handle local sensing and first-hop relaying while H-UAVs manage regional coordination and long-range backhaul transmission, enables scalable, resilient, and information freshness-aware data collection in large-scale WSNs.

### 3.1. Heterogeneous UAV-Assisted Uplink Data Transmissions

The data collection process within the proposed heterogeneous UAV-assisted WSN operates in a hierarchical and time-slotted manner. Each time slot *t* of duration Δt is divided into two distinct sub-slots $\tau_{1}$ and $\tau_{2}$, satisfying $\tau_{1}+\tau_{2}=\Delta t$. This temporal division enables a two-stage data transmission process: local collection within a partition during $\tau_{1}$, followed by coordinated aggregation and forwarding from H-UAVs to the RAP during $\tau_{2}$. To mitigate inter-cell interference, orthogonal channels are employed among the different power-Voronoi partitioned regions, allowing for independent operation within each region $V_{k}\left(t\right)$ during the data collection and initial aggregation phases.

#### 3.1.1. Channel Model

The communication links within the network are modeled using a combination of path loss and small-scale fading. Let $d_{ab}\left(t\right)$ denote the Euclidean distance between any two nodes *a* and *b* at time slot *t*. The channel gain $h_{ab}\left(t\right)$ between nodes *a* and *b* is given by:

(1) $$\begin{array}{c} h_{ab}\left(t\right)=\sqrt{\alpha_{1}d_{ab}^{-\alpha_{0}}\left(t\right)}{\tilde{h}}_{ab}\left(t\right), \end{array}$$

where $\alpha_{0}$ is the path loss exponent characterizing the rate of signal attenuation over distance, $\alpha_{1}$ is a constant incorporating antenna gains and the reference path loss at unit distance, and ${\tilde{h}}_{ab}\left(t\right)∼\mathrm{CN}\left(0,1\right)$ represents the complex Gaussian random variable modeling Rayleigh small-scale fading with zero mean and unit variance. Specifically, $h_{ij}\left(t\right)$ denotes the channel gain between GU $i\in N_{G}$ and L-UAV $j\in N_{L}$, $h_{jk}\left(t\right)$ is the channel gain between L-UAV $j\in N_{L}$ and its associated H-UAV $k\in N_{H}$, and $h_{k0}\left(t\right)$ indicates the channel gain between H-UAV $k\in N_{H}$ and the RAP located at a fixed position $r_{0}$.

#### 3.1.2. GU-to-L-UAV Transmissions in Sub-Slot $\tau_{1}$

During the first sub-slot $\tau_{1}$, each L-UAV *j* within a partition $V_{k}\left(t\right)$ establishes a connection with a single associated GU $i\in L_{k}^{g}$. The received signal at L-UAV *j* from its associated GU *i* is subject to interference from other active GUs within the same partition $V_{k}\left(t\right)$. The signal-to-interference-plus-noise ratio (SINR) for the link from GU *i* to L-UAV *j* is expressed by:

(2) $$\begin{array}{c} \gamma_{ij}^{\left(1\right)}\left(t\right)=\frac{P_{i}^{g}{\left|h_{ij}\left(t\right)\right|}^{2}}{\sigma^{2}+\sum_{i^{\prime }\in L_{k}^{g}\backslash \left\{i\right\}}P_{i^{\prime }}^{g}{\left|h_{i^{\prime }j}\left(t\right)\right|}^{2}}, \end{array}$$

where $P_{i}^{g}$ is the transmit power of GU *i*, $\sigma^{2}$ is the power of the additive white Gaussian noise (AWGN) at the L-UAV receiver, and the summation term represents the aggregate interference power from all other active GUs $i^{\prime }$ within the same partition $V_{k}\left(t\right)$, excluding the desired signal from GU *i*. The achievable data rate for the transmission from GU *i* to its associated L-UAV *j* during sub-slot $\tau_{1}$ is calculated as:

(3) $$\begin{array}{c} R_{ij}^{\left(1\right)}\left(t\right)=\tau_{1}B\log_{2}\left(1+\gamma_{ij}^{\left(1\right)}\left(t\right)\right), \end{array}$$

where *B* represents the system bandwidth.

#### 3.1.3. NOMA Uplink from L-UAVs to H-UAVs in Sub-Slot $\tau_{2}$

During the second sub-slot $\tau_{2}$, L-UAVs within each partition $V_{k}\left(t\right)$ simultaneously transmit their collected data to their associated H-UAV *k* using NOMA to efficiently utilize the available spectrum and handle multiple concurrent transmissions. The H-UAV *k* operates in full-duplex mode, receiving data from its associated L-UAVs while simultaneously forwarding aggregated data to the RAP using OFDMA. The received signal $y_{k}\left(t\right)$ at H-UAV *k* is formed by the superposition of signals from all L-UAVs within its associated partition $V_{k}\left(t\right)$, denoted by the set $L_{k}^{l}$, and is given by:

(4) $$\begin{array}{c} y_{k}\left(t\right)=\sum_{j\in L_{k}^{l}}h_{jk}\left(t\right)\sqrt{P_{j}^{l}}x_{j}\left(t\right)+\eta_{SI}\left(t\right)+n_{k}\left(t\right), \end{array}$$

where each L-UAV $j\in L_{k}^{l}$ transmits its data symbol $x_{j}\left(t\right)$ with unit average power ($E[|x_{j}\left(t\right){|}^{2}]=1$) and transmit power $P_{j}^{l}$ over a channel characterized by the complex gain $h_{jk}\left(t\right)$. $\eta_{SI}\left(t\right)$ is the residual self-interference arising from the H-UAV’s imperfect full-duplex operation with average power $I_{SI}\left(t\right)=E[|\eta_{SI}\left(t\right){|}^{2}]$, and $n_{k}\left(t\right)∼\mathrm{CN}\left(0,\sigma^{2}\right)$ is the AWGN at the receiver.

To decode the superimposed signals, H-UAV *k* employs successive interference cancellation (SIC). The SIC decoding order is determined by the channel conditions, typically sorted by the squared channel gains ${\left|h_{jk}\left(t\right)\right|}^{2}$. Without loss of generality, the L-UAVs in $L_{k}^{l}$ are ordered as ${\left|h_{1k}\left(t\right)\right|}^{2}\geq {\left|h_{2k}\left(t\right)\right|}^{2}\geq \cdots \geq {\left|h_{|L_{k}^{l}|k}\left(t\right)\right|}^{2}$. Assuming successful cancellation of stronger signals, the SINR for decoding the signal from L-UAV *j* is given by:

(5) $$\begin{array}{c} \gamma_{jk}^{\left(2\right)}\left(t\right)=\frac{P_{j}^{l}{\left|h_{jk}\left(t\right)\right|}^{2}}{\sigma^{2}+I_{SI}\left(t\right)+\sum_{m\in L_{k}^{l},m>j}P_{m}^{l}{\left|h_{mk}\left(t\right)\right|}^{2}}. \end{array}$$

The achievable data rate for L-UAV *j*’s transmission to H-UAV *k* during sub-slot $\tau_{2}$ is:

(6) $$\begin{array}{c} R_{j}^{\left(2\right)}\left(t\right)=\tau_{2}B\log_{2}\left(1+\gamma_{jk}^{\left(2\right)}\left(t\right)\right). \end{array}$$

Then, the sum-throughput for the NOMA uplink from all L-UAVs in partition $V_{k}\left(t\right)$ to H-UAV *k* during $\tau_{2}$ is $R_{k}^{\mathrm{NOMA}}\left(t\right)=\sum_{j\in L_{k}^{l}}R_{j}^{\left(2\right)}\left(t\right)$.

Note that we adopt a standard abstraction for NOMA with SIC: decoding is assumed successful whenever the SINR constraints are satisfied. This yields a tractable cross-layer model that couples AoI evolution with queue dynamics and PHY-layer link budgets. The main non-ideal effect explicitly captured in the full-duplex relay is the residual self-interference term. In practice, SIC may be imperfect due to channel estimation errors, finite blocklength, synchronization mismatch, and hardware limitations, leading to decoding errors and residual multi-user interference even when SINR thresholds are met. Imperfect SIC or packet drops reduce the service success probability of each transmission attempt, lowering the effective queue departure rate and increasing backlog, waiting time, and thus average AoI. A straightforward extension is to replace the deterministic SINR threshold with a stochastic service model with success probability $p_{\mathrm{succ}}\left(\mathrm{SINR}\right)$, and to model residual post-SIC interference by adding ηI with η∈[0,1] to the interference power. In this case, failed packets remain in the buffer, and the AoI/queue update equations remain valid with probabilistic departures. Although such non-idealities would increase the absolute AoI, our proposed AoI-aware hierarchical MADRL framework is expected to retain its advantage because trajectory control and adaptive partitioning improve link quality and load balancing, yielding higher effective success probability and reduced congestion even under imperfect SIC. For simplicity, we retain the standard NOMA-with-SIC abstraction in this work.

#### 3.1.4. OFDMA Downlink from H-UAVs to RAP in Sub-Slot $\tau_{2}$

During the second sub-slot $\tau_{2}$, the full-duplex H-UAV *k* simultaneously transmits the aggregated data collected from its associated L-UAVs to the RAP. To ensure efficient and interference-free communication from multiple H-UAVs to the RAP, OFDMA is employed. The SINR for the link from H-UAV *k* to the RAP is given by:

(7) $$\begin{array}{c} \gamma_{k0}\left(t\right)=\frac{P_{k}^{h}{\left|h_{k0}\left(t\right)\right|}^{2}}{\sigma^{2}}, \end{array}$$

where $P_{k}^{h}$ is the transmit power allocated by H-UAV *k* for the downlink transmission to the RAP, and ${\left|h_{k0}\left(t\right)\right|}^{2}$ represents the squared magnitude of the channel gain between H-UAV *k* and the RAP at time *t*. The data rate for the transmission from H-UAV *k* to the RAP during sub-slot $\tau_{2}$ is:

(8) $$\begin{array}{c} R_{k0}\left(t\right)=\tau_{2}B\log_{2}\left(1+\gamma_{k0}\left(t\right)\right), \end{array}$$

and the total throughput achieved at the RAP, which aggregates data from all H-UAVs, is $R_{0}\left(t\right)=\sum_{k\in N_{H}}R_{k0}\left(t\right)$. This total throughput represents the system’s end-to-end data delivery capability to the central access point and serves as a key performance metric for evaluating the efficiency of the proposed heterogeneous UAV-assisted data collection framework.

### 3.2. Flow Conservation and Data Queue Dynamics

To model the end-to-end data flow and buffer management in the heterogeneous UAV network, we define the queue dynamics at both L-UAVs and H-UAVs, capturing the storage and forwarding processes that ensure data conservation across the multi-tier architecture. Let $Q_{j}^{\left(L\right)}\left(t\right)$ denote the data queue length (in bits) at L-UAV *j* at the beginning of time slot *t*, and let $Q_{k}^{\left(H\right)}\left(t\right)$ represent the queue length at H-UAV *k*. The queue evolution for L-UAV *j*, which is associated with GU *i* and serves as a relay to H-UAV *k*, is governed by:

(9) $$\begin{array}{c} Q_{j}^{\left(L\right)}\left(t+1\right)=\max\left\{\min\left\{Q_{j}^{\left(L\right)}\left(t\right)+R_{ij}^{\left(1\right)}\left(t\right)-R_{j}^{\left(2\right)}\left(t\right),Q_{\max}\right\},0\right\}, \end{array}$$

where data is accumulated during $\tau_{1}$ via the GU-to-L-UAV link at rate $R_{ij}^{\left(1\right)}\left(t\right)$, and then transmitted and removed from the queue during $\tau_{2}$ at rate $R_{j}^{\left(2\right)}\left(t\right)$; $Q_{\max}$ denotes the maximum buffer capacity. Similarly, the queue dynamics for H-UAV *k* are described by:

(10) $$\begin{array}{c} Q_{k}^{\left(H\right)}\left(t+1\right)=\max\left\{\min\left\{Q_{k}^{\left(H\right)}\left(t\right)+R_{k}^{\mathrm{NOMA}}\left(t\right)-R_{k0}\left(t\right),Q_{\max}\right\},0\right\}, \end{array}$$

capturing the aggregation of data from multiple L-UAVs at rate $R_{k}^{\mathrm{NOMA}}\left(t\right)$ and its subsequent forwarding to the RAP at rate $R_{k0}\left(t\right)$.

### 3.3. AoI Dynamics with L-UAV and H-UAV Queueing Under Full-Duplex Relaying

To accurately reflect the impact of finite-rate links and multi-hop buffering on information freshness, we extend the AoI model to incorporate queuing delays at both L-UAV and H-UAV buffers, leveraging the full-duplex capability of H-UAVs. We introduce the scheduling variable $\beta_{ij}\left(t\right)\in \left\{0,1\right\}$, indicating whether GU *i* transmits its status update (of size $B_{i}\left(t\right)$ bits) to L-UAV *j* in sub-slot $\tau_{1}$. Each GU $i\in L_{k}^{g}$ belongs to region $V_{k}\left(t\right)$ served by L-UAVs $L_{k}^{l}$, with the association constraint:

(11) $$\sum_{j\in L_{k}^{l}}\beta_{ij}\left(t\right)\leq 1,\;\forall i\in L_{k}^{g},$$

which ensures that each GU is associated with at most one L-UAV per time slot.

When $\beta_{ij}\left(t\right)=1$, the packet traverses three stages. In Stage 1 (GU to L-UAV), the packet is transmitted at rate $R_{ij}^{\left(1\right)}\left(t\right)$ over duration $\tau_{1}$. Successful reception requires $R_{ij}^{\left(1\right)}\left(t\right)\geq \frac{B_{i}\left(t\right)}{\tau_{1}}$, and if satisfied along with the buffer constraint $Q_{j}^{\left(L\right)}\left(t\right)+B_{i}\left(t\right)\leq Q_{\max}$, the packet enters L-UAV *j*’s queue (otherwise it is dropped). In Stage 2 (L-UAV to H-UAV), during $\tau_{2}$, L-UAV *j* transmits to H-UAV *k* at rate $R_{j}^{\left(2\right)}\left(t\right)$. The packet can be dequeued only if $R_{j}^{\left(2\right)}\left(t\right)\geq \frac{B_{i}\left(t\right)}{\tau_{2}}$ and the H-UAV buffer has space ($Q_{k}^{\left(H\right)}\left(t\right)+B_{i}\left(t\right)\leq Q_{\max}$). In Stage 3 (H-UAV to RAP), thanks to full-duplex operation, H-UAV *k* simultaneously receives from L-UAVs and forwards aggregated data to the RAP via OFDMA. Successful decoding at the RAP requires the SNR $\gamma_{k0}\left(t\right)\geq \gamma_{0}$. Let $g_{i}\left(t\right)\in \left\{0,1\right\}$ indicate whether the packet transmitted by GU *i* was generated in slot *t*. Given successful completion of all stages, the total latency of GU *i*’s packet is:

(12) $$D_{i}\left(t\right)=g_{i}\left(t\right)\left(\tau_{1}+\frac{B_{i}\left(t\right)}{R_{k0}\left(t\right)}\right)+\frac{Q_{j}^{\left(L\right)}\left(t\right)}{R_{j}^{\left(2\right)}\left(t\right)}+\frac{Q_{k}^{\left(H\right)}\left(t\right)}{R_{k0}\left(t\right)},$$

where the first term captures the transmission time for a fresh packet (if $g_{i}\left(t\right)=1$), the second term accounts for waiting time in the L-UAV queue, and the third term represents waiting time in the H-UAV queue for all data ahead of GU *i*’s packet (including packets from other L-UAVs scheduled in the same or earlier slots). Note that $R_{k0}\left(t\right)$ is the effective service rate from H-UAV *k* to the RAP. This refined AoI model explicitly couples information freshness to physical-layer rates, MAC-layer scheduling, and network-layer queuing, enabling holistic design of mobility, resource allocation, and data collection policies under realistic full-duplex H-UAV relaying.

The end-to-end success indicator $s_{i}\left(t\right)\in \left\{0,1\right\}$ is defined as:

(13) $$s_{i}\left(t\right)=\sum_{j\in L_{k}^{l}}\beta_{ij}\left(t\right)\cdot I\left[R_{ij}^{\left(1\right)}\left(t\right)\geq \frac{B_{i}\left(t\right)}{\tau_{1}}\right]\cdot I\left[R_{j}^{\left(2\right)}\left(t\right)\geq \frac{B_{i}\left(t\right)}{\tau_{2}}\right]\cdot I\left[\gamma_{k0}\left(t\right)\geq \gamma_{0}\right],$$

where I[·] is the indicator function. The AoI for GU *i* evolves as:

(14) $$\Delta_{i}\left(t+1\right)=\left(1-s_{i}\left(t\right)\right)\left(\Delta_{i}\left(t\right)+\Delta t\right)+s_{i}\left(t\right)D_{i}\left(t\right).$$

This model fully captures the interplay between scheduling, physical-layer rates, and two-tier queuing under full-duplex H-UAV relaying, enabling precise AoI-aware control of UAV trajectories, user association, and resource allocation while respecting practical buffer and latency constraints.

## 4. AoI-Aware Hierarchical MADRL for Coordinated Coverage and Collection with Hybrid UAV Swarms

The hierarchical architecture decomposes the global data collection task into two coupled but computationally separable subproblems: regional coordination and trajectory optimization for H-UAVs, and local mobility and transmission control for L-UAVs. This decomposition enables a scalable solution framework that combines multi-agent deep reinforcement learning (MADRL) for high-level strategic planning with distributed reactive control for low-level execution. In this section, we formulate the AoI minimization problem, characterize the dynamic power-Voronoi partitioning mechanism, present the weighted Vicsek-based mobility control for L-UAVs, and develop a digital twin-enhanced MADRL approach for H-UAV trajectory planning and resource allocation. The proposed framework explicitly couples information freshness metrics with physical-layer communication constraints, queue dynamics, and multi-agent coordination under uncertainty.

### 4.1. AoI Minimization Problem Formulation

The optimization objective is to minimize the time-averaged sum-AoI across all GUs over the operational horizon, subject to constraints on UAV mobility, power budgets, queue stability, collision avoidance, and communication feasibility. The problem is then formulated as:

(15a)minimizeA(t)1TNg∑t=0T−1∑i∈NGΔi(t)(15b)s.t.uk(t+Δt)=uk(t)+vk(t)Δt,∀k∈NH,(15c)vk(t+Δt)=vk(t)+Δuk(t),∥vk(t)∥≤vmaxH,∀k∈NH,(15d)∥Δuk(t)∥≤amaxHΔt,∀k∈NH,(15e)pl(t+Δt)=pl(t)+vl(t)Δt,pl(t)∈Vk(t),∀l∈Lkl,k∈NH,(15f)∥vl(t)∥=v0,∀l∈NL,(15g)∥uk(t)−uj(t)∥≥dminHH,∀k≠j∈NH,(15h)∥pl(t)−pm(t)∥≥dminLL,∀l≠m∈NL,(15i)Qj(L)(t+1)=maxminQj(L)(t)+Rij(1)(t)−Rj(2)(t),Qmax,0,(15j)Qk(H)(t+1)=maxminQk(H)(t)+RkNOMA(t)−Rk0(t),Qmax,0,(15k)∑j∈Lklβij(t)≤1,∀i∈Lkg.

Here, $A\left(t\right)=\{A_{H}\left(t\right),A_{L}\left(t\right)\}$, where $A_{H}\left(t\right)={\left\{a_{k}\left(t\right)\right\}}_{k\in N_{H}}$ with $a_{k}\left(t\right)=\left\{\Delta u_{k}\left(t\right)\right\}$ specifies the H-UAV’s velocity increment, and $A_{L}\left(t\right)=\left\{\alpha_{\mathrm{AoI}},\alpha_{\mathrm{link}},\alpha_{\mathrm{cong}},\alpha_{\mathrm{bdry}}\right\}$ denotes the weight parameters governing the Vicsek-based mobility model for L-UAVs, which can be adapted spatially or temporally. Constraint Equation (15b) enforces the kinematic relationship between H-UAV position and velocity, while Equation (15c) and Equation (15d) bound the maximum velocity $v_{\max}^{H}$ and acceleration $a_{\max}^{H}$ of H-UAVs, respectively. Constraint Equation (15e) ensures that L-UAVs remain within their assigned power-Voronoi cells $V_{k}\left(t\right)$ and evolve according to the weighted Vicsek model, with constant speed $v_{0}$ enforced by Equation (15f). Collision avoidance among H-UAVs and L-UAVs is guaranteed by Equation (15g) and Equation (15h), requiring minimum inter-UAV separations $d_{\min}^{HH}$ and $d_{\min}^{LL}$, respectively.

The formulated problem Equation (15) yields a large-scale, nonconvex, mixed-integer stochastic optimization problem characterized by high-dimensional state-action spaces, strongly coupled spatiotemporal dynamics, partial observability, and nonstationary environments due to time-varying channel conditions and traffic patterns. These challenges render conventional optimization methods intractable for real-time deployment, thereby motivating a learning-based hierarchical solution.

### 4.2. Power-Voronoi Partitioning with Adaptive Weights

To ensure balanced and dynamic task allocation among the H-UAVs, the operational area Ω is partitioned into $K=|N_{H}|$ non-overlapping regions using a power-Voronoi diagram with adaptive weights. This approach allows the system to flexibly adjust the coverage responsibilities of each H-UAV based on real-time network conditions, including the density of data generation, the distribution of L-UAVs, and the queue status of the H-UAVs themselves.

The power-Voronoi cell $V_{k}\left(t\right)$ associated with H-UAV *k* at time *t* is defined as the set of all points x∈Ω for which the weighted distance to the position of H-UAV *k*, denoted by $u_{k}\left(t\right)$, is less than or equal to the weighted distance to any other H-UAV $j\in N_{H}\backslash \left\{k\right\}$. The weight $w_{k}\left(t\right)$ associated with each H-UAV plays a crucial role in shaping the cell boundaries. Formally, the cell $V_{k}\left(t\right)$ is defined by

(16) $$\begin{array}{c} V_{k}\left(t\right)=\left\{x\in \Omega \;:\;∥x-u_{k}\left(t\right)∥-w_{k}\left(t\right)\leq ∥x-u_{j}\left(t\right)∥-w_{j}\left(t\right),\;\forall j\in N_{H}\backslash \left\{k\right\}\right\}, \end{array}$$

where ∥·∥ denotes the Euclidean norm. The term $∥x-u_{k}(t)∥-w_{k}(t)$ represents the power distance from point *x* to H-UAV *k*. When all weights $w_{k}\left(t\right)$ are zero, the power-Voronoi diagram reduces to the standard Voronoi diagram. By adjusting the weights $w_{k}\left(t\right)$, the size and shape of the cells $V_{k}\left(t\right)$ can be dynamically controlled to reflect the operational state of each region.

The adaptive weight $w_{k}\left(t\right)$ for H-UAV *k* at time *t* is calculated to reflect the current load and operational state of its associated region $V_{k}\left(t\right)$. The weight for the next time slot, $w_{k}\left(t+1\right)$, is computed using a convex combination of three key network state metrics: the data generation density within the cell, the density of L-UAVs, and the queue length at the H-UAV. This calculation is performed at the beginning of each time slot and is given by

(17) $$w_{k}\left(t+1\right)=\alpha \frac{\Lambda_{k}\left(t\right)}{U_{k}\left(t\right)}+\beta \frac{\left|L_{k}^{l}\left(t\right)\right|}{U_{k}\left(t\right)}+\gamma \frac{Q_{k}^{H}\left(t\right)}{Q_{max}},$$

where $\Lambda_{k}\left(t\right)=\sum_{i\in L_{k}^{g}}\lambda_{i}\left(t\right)$ is the total data generation rate within cell $V_{k}\left(t\right)$, representing the data generation density. $L_{k}^{g}$ denotes the set of GUs located within $V_{k}\left(t\right)$ and $\lambda_{i}\left(t\right)$ is the instantaneous data generation rate of GU *i*. $U_{k}\left(t\right)$ is the area of the power-Voronoi cell $V_{k}\left(t\right)$, so that $\frac{\Lambda_{k}\left(t\right)}{U_{k}\left(t\right)}$ provides a normalized measure of the data load per unit area. $\left|L_{k}^{l}\left(t\right)\right|$ is the number of L-UAVs present in cell $V_{k}\left(t\right)$ at time *t*, and the ratio $\frac{\left|L_{k}^{l}\left(t\right)\right|}{U_{k}\left(t\right)}$ represents the L-UAV density within the cell. A higher density indicates a region already well-served by L-UAVs, potentially requiring less coverage area, hence a higher weight to shrink the cell. The last term is the normalized queue length at H-UAV *k*, reflecting its current data processing burden. A larger queue suggests that the H-UAV is struggling to offload data, potentially requiring a smaller service area, thus a higher weight. α, β, γ are positive weighting coefficients satisfying α+β+γ=1, determining the relative importance of data generation density, L-UAV density, and H-UAV queue length, respectively. These coefficients can be tuned based on system priorities, e.g., AoI minimization, load balancing, or energy efficiency. The adaptive weight $w_{k}\left(t+1\right)$ is then used in the power-Voronoi partitioning for the subsequent time slot t+1.

This adaptive power-Voronoi partitioning scheme offers several significant advantages for the heterogeneous UAV system. It facilitates dynamic load balancing by incorporating real-time data generation rates and H-UAV queue lengths into the weight calculation, allowing the partitioning to automatically adjust and shift coverage responsibilities from heavily loaded H-UAVs to those with lighter workloads, thereby preventing bottlenecks and promoting system equilibrium. The scheme demonstrates inherent scalability, naturally accommodating fluctuations in the number of both H-UAVs and GUs without requiring fundamental changes to the partitioning algorithm. Its flexibility is achieved through the convex combination used in the weight calculation, which permits fine-tuning of the partitioning behavior via adjustments to the parameters α, β, and γ, allowing the system to prioritize specific metrics as needed. Finally, the dynamic nature of the evolving partition shapes provides a valuable reference for H-UAV trajectory planning, ensuring that H-UAVs can strategically position themselves to effectively serve their assigned regions, thus enhancing overall coverage and data collection efficiency. This adaptive partitioning strategy forms the foundation for the coordinated coverage and data collection framework, enabling efficient resource management and scalability in the heterogeneous UAV-assisted WSN.

### 4.3. Weighted Vicsek Model for L-UAV Mobility

To enable self-organized, region-constrained motion that jointly optimizes information freshness, link reliability, and spatial safety, we propose a weighted Vicsek-inspired velocity update rule for each L-UAV $l\in L_{k}^{l}$ within its assigned power-Voronoi region $V_{k}\left(t\right)$. The controller balances four objectives: proximity to high-AoI ground users to reduce staleness, favorable channel conditions for both GU-to-L-UAV and L-UAV-to-H-UAV links, inter-UAV decongestion for collision avoidance and coverage diversity, and confinement within the designated service region.

At each time slot *t*, L-UAV *l* updates its velocity and position according to

(18) $$v_{l}\left(t+\Delta t\right)=v_{0}\;\frac{u_{l}\left(t\right)}{\max\{∥u_{l}\left(t\right)∥,\epsilon \}},\;p_{l}\left(t+\Delta t\right)=p_{l}\left(t\right)+v_{l}\left(t+\Delta t\right)\;\Delta t,$$

where $v_{0}$ is the constant speed magnitude, ϵ>0 prevents division by zero, and the unnormalized direction vector $u_{l}\left(t\right)$ aggregates alignment and task-specific forces as

(19) $$u_{l}\left(t\right)=\sum_{j\in L_{k}^{l}\left(t\right)}w_{lj}\;\frac{v_{j}\left(t\right)}{∥v_{j}\left(t\right)∥}+f_{l}^{\mathrm{AoI}}\left(t\right)+f_{l}^{\mathrm{link}}\left(t\right)+f_{l}^{\mathrm{cong}}\left(t\right)+f_{l}^{\mathrm{bdry}}\left(t\right),$$

where the first term represents Vicsek alignment with neighboring L-UAVs weighted by $w_{lj}\geq 0$ (normalized such that $\sum_{j\in L_{k}^{l}\left(t\right)}w_{lj}=1$), optionally incorporating neighbor reliability or link quality, and the subsequent terms are task-specific forces described below.

Let $M_{l}\left(t\right)\subseteq G_{k}$ be the set of GUs within sensing radius $r_{s}$ of L-UAV *l*. To prioritize stale yet feasibly servable users, we define an AoI-weighted attraction force. The AoI weight for GU *i* is computed as

(20) $$\omega_{i}^{\mathrm{AoI}}\left(t\right)=\alpha_{\mathrm{AoI}}\;\frac{\Delta_{i}\left(t\right)-\Delta_{\min}}{\Delta_{\max}-\Delta_{\min}}I\left[\gamma_{il}^{\left(1\right)}\left(t\right)-\gamma_{1}\right],$$

where $\Delta_{i}\left(t\right)$ is the AoI of GU *i* (as defined in Section 3.3) normalized between $\Delta_{\min}$ and $\Delta_{\max}$, and the second factor is a GU-to-L-UAV link quality score ensuring that only GUs with decodable links ($\gamma_{il}^{\left(1\right)}\left(t\right)\geq \gamma_{1}$) in sub-slot $\tau_{1}$ exert influence. The resulting force pulls L-UAV *l* toward high-priority GUs:

(21) $$f_{l}^{\mathrm{AoI}}\left(t\right)=\sum_{i\in M_{l}\left(t\right)}\omega_{i}^{\mathrm{AoI}}\left(t\right)\;\frac{p_{i}-p_{l}\left(t\right)}{∥p_{i}-p_{l}\left(t\right)∥}.$$

The AoI-driven attraction force $f_{l}^{\mathrm{AoI}}\left(t\right)$, which directs L-UAV *l* toward GUs with high normalized AoI that are feasibly servable. The $\omega_{i}^{\mathrm{AoI}}\left(t\right)$ defined in Equation (20) ensures that the force magnitude is proportional to both information staleness and channel quality, preventing L-UAVs from being attracted to high-AoI GUs that are too distant or obstructed to serve effectively. This coupling between AoI and physical-layer feasibility is central to the proposed framework: by explicitly incorporating SINR-based link quality into the mobility controller, the Vicsek model becomes sub-slot aware, dynamically adapting L-UAV trajectories to the instantaneous communication environment and thereby maximizing the probability of successful packet reception (as indicated by the success criterion $s_{i}\left(t\right)$ in Equation (13)).

To enhance the second-hop uplink during sub-slot $\tau_{2}$, L-UAV *l* is attracted toward its serving H-UAV *k* based on the quality of the L-UAV-to-H-UAV NOMA channel. The link quality weight is computed as

(22) $$\omega_{l}^{\mathrm{link}}\left(t\right)=\alpha_{\mathrm{link}}\;I\left[\gamma_{lk}^{\left(2\right)}\left(t\right)-\gamma_{2}\right]\frac{1}{1+\eta_{\mathrm{order}}\left(u_{l}-1\right)},$$

where $u_{l}$ is the decoding index of L-UAV *l* in the SIC sequence of H-UAV *k*, and $\eta_{\mathrm{order}}\geq 0$ optionally down-weights late-decoded users to account for SIC ordering. The corresponding force aligns *l* with the LOS direction to H-UAV *k*:

(23) $$f_{l}^{\mathrm{link}}\left(t\right)=\omega_{l}^{\mathrm{link}}\left(t\right)\;\frac{p_{k}\left(t\right)-p_{l}\left(t\right)}{∥p_{k}\left(t\right)-p_{l}\left(t\right)∥}.$$

The link quality enhancement force $f_{l}^{\mathrm{link}}\left(t\right)$ complements the GU-attraction mechanism by pulling L-UAV *l* toward its serving H-UAV *k* when the uplink SINR $\gamma_{lk}^{\left(2\right)}\left(t\right)$ is marginal, thereby improving the second-hop relay link during sub-slot $\tau_{2}$.

To maintain safe separation and promote spatial diversity, a repulsive force acts on neighbors within exclusion radius $r_{\min}$:

(24) $$f_{l}^{\mathrm{cong}}\left(t\right)=-\alpha_{\mathrm{cong}}\sum_{j\in L_{k}^{l}\left(t\right)}\frac{1}{{∥p_{j}\left(t\right)-p_{l}\left(t\right)∥}^{2}}\;\frac{p_{j}\left(t\right)-p_{l}\left(t\right)}{∥p_{j}\left(t\right)-p_{l}\left(t\right)∥},$$

The congestion avoidance force $f_{l}^{\mathrm{cong}}\left(t\right)$ maintains safe separation $d_{\min}^{LL}$ among L-UAVs, preventing clustering that would cause excessive co-channel interference in the GU-to-L-UAV uplink and ensuring spatial diversity for coverage.

To enforce region assignment, a soft boundary force is applied when L-UAV *l* approaches the boundary $\partial V_{k}$ of its assigned region:

(25) $$f_{l}^{\mathrm{bdry}}\left(t\right)=\left\{\begin{array}{cc} -\alpha_{\mathrm{bdry}}\;\nabla_{p_{l}}\left[d{\left(p_{l}\left(t\right),\partial V_{k}\right)}^{-1}\right], & \mathrm{if}\;d\left(p_{l}\left(t\right),\partial V_{k}\right)<r_{b},\\ 0, & \mathrm{otherwise}, \end{array}\right.$$

where $d(p_{l}\left(t\right),\partial V_{k})$ is the distance from L-UAV *l* to the boundary and $r_{b}$ is a threshold distance. Equivalently, the updated position $p_{l}\left(t+\Delta t\right)$ can be projected onto the closest point inside $V_{k}$ to ensure hard constraint enforcement. The boundary confinement force $f_{l}^{\mathrm{bdry}}\left(t\right)$ enforces the constraint $p_{l}\left(t\right)\in V_{k}\left(t\right)$ by applying a repulsive gradient when L-UAV *l* approaches the cell boundary $\partial V_{k}\left(t\right)$, or equivalently by projecting the updated position onto the interior of $V_{k}\left(t\right)$, thereby respecting the territorial assignments induced by the power-Voronoi partition.

The gains $\alpha_{\mathrm{AoI}},\alpha_{\mathrm{link}},\alpha_{\mathrm{cong}},\alpha_{\mathrm{bdry}}$, thresholds $\gamma_{1},\gamma_{2}$, and smoothing parameters $\delta_{1},\delta_{2},\epsilon_{c}$ provide tunable trade-offs between responsiveness and stability. Crucially, the mobility law is sub-slot aware, leveraging real-time SINR estimates from both transmission phases and thereby coupling UAV motion directly to end-to-end service feasibility and, through the AoI dynamics in Equation (14), to long-term information freshness. This formulation enables L-UAV swarms to autonomously reconfigure toward regions of high information staleness while avoiding collisions and respecting territorial assignments.

The L-UAV tier operates in a fully decentralized manner, executing local decisions via the weighted Vicsek model. Each L-UAV $l\in L_{k}^{l}$ updates its velocity according to Equations (18) and (19), aggregating alignment with neighbors, AoI-driven attraction toward stale GUs, link-quality enhancement toward its serving H-UAV, congestion avoidance, and boundary confinement. This design ensures scalability to large-scale agents while maintaining real-time responsiveness. Notably, the mobility controller leverages instantaneous SINR estimates from both transmission phases ($\tau_{1}$ and $\tau_{2}$) to couple motion directly to end-to-end service feasibility. During $\tau_{1}$, each L-UAV greedily associates with the GU in its sensing radius that maximizes an AoI-channel utility metric; during $\tau_{2}$, all L-UAVs in $L_{k}^{l}$ transmit simultaneously to H-UAV *k* using NOMA. This two-phase protocol minimizes coordination overhead while preserving performance.

The weighted Vicsek-inspired controller plays a central role in coordinating the weak agents (L-UAVs). Here, we further summarize the main mechanisms in our design that mitigate oscillations, excessive clustering, and deadlock, especially under dynamically evolving (power-)Voronoi partitions.

- Bounded updates and numerical robustness: Our L-UAV motion update follows a bounded-step direction-field form, where the speed is fixed (or upper-bounded) and only the heading is updated using the normalized resultant vector. Concretely, with a normalization term $\max\{∥u_{l}\left(t\right)∥,\epsilon \}$ (for a small ϵ>0), the update prevents unbounded accelerations and improves numerical robustness. This boundedness inherently limits abrupt changes in motion and reduces high-frequency oscillations.
- L-UAV swarm avoids excessive clustering: The resultant control vector is composed of complementary terms. In particular, the congestion avoidance force term $f_{l}^{\mathrm{cong}}\left(t\right)$ introduces short-range repulsion among nearby L-UAVs, acting as a soft separation constraint. This mechanism prevents excessive clustering and alleviates local deadlock caused by overcrowding in the same area. In practice, the repulsion magnitude can be clipped to avoid overly stiff responses that may induce jitter.
- Feasibility gating reduces futile oscillations: The task-driven attraction components (e.g., toward high-AoI regions or relay opportunities) are gated by link feasibility indicators (such as SINR/connectivity conditions). Hence, targets that are temporarily unreachable do not generate attraction, which avoids chasing behaviors and reduces oscillations due to repeatedly switching to infeasible objectives.
- Handling dynamic Voronoi partitions: When Voronoi regions evolve due to H-UAV decisions, boundary movement can in principle cause chattering near partition edges. Our design addresses this in two ways. First, the boundary-keeping term $f_{l}^{\mathrm{bdry}}\left(t\right)$ is activated only within a buffer distance from the boundary, which introduces hysteresis and reduces sensitivity to small boundary shifts. Moreover, if an L-UAV approaches or crosses the boundary, a projection step keeps the position within the feasible region, guaranteeing region adherence. Second, an implementation-friendly time-scale separation is adopted. Partition weights (and thus Voronoi boundaries) are updated at a slower period by H-UAVs than the L-UAV heading updates, or smoothed over time. This reduces high-frequency boundary fluctuations and improves stability without changing the overall framework.

While a complete closed-form stability proof is beyond the scope of this work, the above mechanisms are aligned with well-established results and practices in flocking/consensus and potential-field-based multi-robot control. Empirically stable operation is expected when: (i) the neighbor interaction graph is sufficiently connected over time (given sensing/communication radius and agent density), (ii) repulsion/boundary gains are strong enough to prevent collisions and boundary crossing but not so strong that they dominate alignment, (iii) partition updates are not excessively fast (via slower updates or smoothing), and (iv) stochastic disturbances/noise remain within a moderate range.

### 4.4. H-UAVs’ Trajectory Planning via MADDPG

#### 4.4.1. POMDP Formulation for H-UAV Coordination

We model the H-UAV coordination problem as a POMDP characterized by the tuple:

(26) $$\langle N_{H},S,{\left\{O_{k}\right\}}_{k\in N_{H}},{\left\{A_{k}\right\}}_{k\in N_{H}},P,{\left\{R_{k}\right\}}_{k\in N_{H}},\gamma_{rl}\rangle ,$$

where S is the global state space, $O_{k}$ is the local observation space of H-UAV *k*, $A_{k}$ is the action space of agent *k*, $P:S\times A_{1}\times \cdots \times A_{N_{h}}\to \Delta \left(S\right)$ is the state transition probability distribution, $R_{k}:S\times A_{1}\times \cdots \times A_{N_{h}}\to R$ is the local reward function for agent *k*, and $\gamma_{rl}\in \left(0,1\right)$ is the discount factor.

The local observation $o_{k}\left(t\right)$ for H-UAV *k* at time *t* includes:

(27) $$\begin{array}{c} o_{k}\left(t\right)=\{u_{k}\left(t\right),v_{k}\left(t\right),Q_{k}^{\left(H\right)}\left(t\right),w_{k}\left(t\right),U_{k}\left(t\right),{\bar{\Delta }}_{k}\left(t\right),\\ \;\;{\bar{\gamma }}_{k}^{\left(2\right)}\left(t\right),\left|L_{k}^{l}\left(t\right)\right|,{\left\{u_{j}\left(t\right),w_{j}\left(t\right)\right\}}_{j\in N_{k}\left(t\right)}\}, \end{array}$$

where $N_{k}\left(t\right)\subset N_{H}\backslash \left\{k\right\}$ denotes neighboring H-UAVs whose cells share a boundary with $V_{k}\left(t\right)$. The observation includes the agent’s own pose $\left(u_{k}\left(t\right),v_{k}\left(t\right)\right)$, queue state $Q_{k}^{\left(H\right)}\left(t\right)$, Voronoi weight $w_{k}\left(t\right)$ and cell area $U_{k}\left(t\right)$, aggregated metrics from the local cell (average AoI of GUs in cell ${\bar{\Delta }}_{k}\left(t\right)=\frac{1}{\left|L_{k}^{g}\left(t\right)\right|}\sum_{i\in L_{k}^{g}\left(t\right)}\Delta_{i}\left(t\right)$, average uplink SINR from associated L-UAVs ${\bar{\gamma }}_{k}^{\left(2\right)}\left(t\right)=\frac{1}{\left|L_{k}^{l}\left(t\right)\right|}\sum_{j\in L_{k}^{l}\left(t\right)}\gamma_{jk}^{\left(2\right)}\left(t\right)$, L-UAV count $\left|L_{k}^{l}\left(t\right)\right|$, and limited state information about neighboring H-UAVs.

The action space $A_{k}$ for H-UAV *k* consists of continuous trajectory control $a_{k}\left(t\right)=\left\{\Delta u_{k}\left(t\right)\right\}$, where $\Delta u_{k}\left(t\right)\in {\left[-a_{\max}^{H}\Delta t,a_{\max}^{H}\Delta t\right]}^{2}$ is the velocity increment that updates $v_{k}\left(t+\Delta t\right)=v_{k}\left(t\right)+\Delta u_{k}\left(t\right)$ subject to $∥v_{k}\left(t+\Delta t\right)∥\leq v_{\max}^{H}$.

The local reward function $R_{k}$ for H-UAV *k* is designed to incentivize AoI reduction within the agent’s region while promoting coordination and constraint satisfaction:

(28) $$\begin{array}{cc} r_{k}\left(t\right)= & -\omega_{1}{\bar{\Delta }}_{k}\left(t\right)-\omega_{2}\max_{i\in L_{k}^{g}\left(t\right)}\Delta_{i}\left(t\right)-\omega_{3}Q_{k}^{\left(H\right)}\left(t\right)\\ \; & +\omega_{4}\sum_{i\in L_{k}^{g}\left(t\right)}s_{i}\left(t\right)-\omega_{5}\sum_{j\in N_{H}\backslash \left\{k\right\}}I\left[∥u_{k}\left(t\right)-u_{j}\left(t\right)∥<d_{\min}^{HH}\right], \end{array}$$

where the first term penalizes the average AoI in cell $V_{k}\left(t\right)$, directly aligning with the global objective Equation (15a); the second term penalizes the peak AoI within the cell, encouraging fairness and preventing the neglect of isolated high-AoI GUs; the third term penalizes queue backlog, promoting proactive data offloading to the RAP and preventing buffer overflow; the fourth term rewards the number of successful end-to-end packet deliveries, directly incentivizing actions that improve link quality and queue service; and the final term imposes a large penalty if H-UAV *k* violates the collision avoidance constraint. The weights ${\left\{\omega_{i}\right\}}_{i=1}^{5}$ are hyperparameters that balance the multiple objectives and can be tuned based on system priorities.

#### 4.4.2. DNN Updates in MADDPG

In the proposed SW-MADRL framework, we employ the multi-agent deep deterministic policy gradient (MADDPG) algorithm to train H-UAVs, which follows a centralized training and decentralized execution paradigm. During the training phase, the critic network serves as a value function estimator. Specifically, the critic network for H-UAV *k*, parameterized by $\phi_{k}$, takes the joint observation $o={\left\{o_{k}\right\}}_{k\in N_{H}}$ and the joint action $a={\left\{a_{k}\right\}}_{k\in N_{H}}$ as inputs. The critic network is updated by minimizing the temporal-difference (TD) loss, which is defined as follows:

(29) $$L_{k}\left(\phi_{k}\right)=E\left[{\left(Q_{k}\left(o,a|\phi_{k}\right)-y_{k}\right)}^{2}\right],$$

where $Q_{k}\left(\cdot \right)$ represents the predicted Q-value, and $y_{k}$ denotes the target value derived from the Bellman equation as follows:

(30) $$y_{k}=r_{k}+\gamma Q_{k}\left(o,a|\phi_{k}\right),$$

where γ∈(0,1) is the discount factor to balance the trade-off between immediate and future rewards.

The actor network $\mu_{k}$, parameterized by $\theta_{k}$, learns the trajectory planning policy for H-UAV *k*. Consistent with the decentralized execution requirement, the actor network generates the action $a_{k}$ based solely on the local observation $o_{k}$. The policy is updated via the deterministic policy gradient to maximize the expected Q-value estimated by the centralized critic as follows:

(31) $$\nabla_{\theta_{k}}J_{k}=E{\left[\nabla_{\theta_{k}}\mu_{k}\left(o_{k}\right)\;\nabla_{a_{k}}Q_{k}\left(o,a\right)\right]}_{a_{k}=\mu_{k}\left(o_{k}\right)}.$$

To improve the stability in learning, both the actor and critic networks maintain their target versions with the parameters $\theta_{k}^{\prime }$ and $\phi_{k}^{\prime }$, respectively, which are updated from the online parameters $\left(\theta_{k},\phi_{k}\right)$ smoothly [29]. This soft update mechanism is defined as:

(32) $$\phi_{k}^{\prime }\leftarrow \tau \phi_{k}+\left(1-\tau \right)\phi_{k}^{\prime },\;\theta_{k}^{\prime }\leftarrow \tau \theta_{k}+\left(1-\tau \right)\theta_{k}^{\prime },$$

where τ∈(0,1) is a small soft update factor.

The complete training procedure is summarized as follows. We first initialize policy networks $\mu_{k}$ and critic networks $Q_{k}$ with random parameters for all H-UAVs *k*, and synchronize their corresponding target networks $\mu_{k}^{\prime }$ and $Q_{k}^{\prime }$ with identical weights. A shared experience replay buffer D is established to store transition data. At the beginning of each episode, the environment is reset. For each time step *t*, each H-UAV *k* observes its local state $o_{k}$ and selects an action $a_{k}=\mu_{k}\left(o_{k}\right)+n$, where n represents exploration noise added to facilitate broad state-space coverage. After executing the joint action a, the agents receive their respective rewards $r_{k}$ and observe the next joint state $o^{\prime }$. The resulting transition tuple $\left(o,a,r,o^{\prime }\right)$ is stored in D. Once sufficient experience is collected, a random mini-batch is sampled from D. The critic network is optimized by minimizing the loss $L_{k}$ based on the target values calculated via the target networks. Subsequently, the actor network is updated using the policy gradient derived from the centralized critic. Finally, the target networks are updated via the soft update mechanism, completing one training iteration.

The computational complexity of the proposed SW-MADRL framework is evaluated as $C_{H}+C_{L}$, where $C_{H}$ and $C_{L}$ denote the individual computational complexities associated with the control of H-UAVs and L-UAVs, respectively. Let $n_{a,f}$ and $n_{c,f}$ denote the number of neurons in the *f*-th layer of the actor and critic networks in MADDPG. Thus, the complexity $C_{H}$ can be expressed as $C_{H}=O\left(N_{h}\left(\sum_{f=0}^{F_{a}-1}n_{a,f}n_{a,f+1}+\sum_{f=0}^{F_{c}-1}n_{c,f}n_{c,f+1}\right)\right)$, where $F_{a}$ and $F_{c}$ represent the total layer number of the actor and critic networks, respectively. Note that the control of L-UAVs is achieved via the weighted Vicsek model, which involves a linear computational process. Therefore, the computational complexity $C_{L}$ is directly proportional to the number of L-UAVs, i.e., $C_{L}=O\left(N_{l}\right)$. Hence, by appropriately adjusting the number of H-UAVs and L-UAVs, a desirable trade-off between system performance and computational complexity can be achieved.

## 5. Numerical Results

In this section, we evaluate the system performance of the SW-MADRL framework. We consider 15 GUs randomly distributed on the ground, with a RAP located at the center. We employ 9 L-UAVs and 3 H-UAVs to support the GUs’ transmissions. The transmit powers of the GUs, L-UAVs, and H-UAVs are set to 20 dBm, 30 dBm, and 40 dBm, respectively. The background noise power is set to −90 dBm. The learning rates of the actor and critic networks are both set to $2\times 10^{-4}$. The other default parameters follow the similar settings in [30]. Both the actor and critic networks are designed as three fully connected layers, where each layer consists of 64 neurons. The replay buffer size is set to $5\times 10^{5}$ and the mini-batch size for training is 256.

### 5.1. Convergence Evaluation of SW-MADRL Framework

**Figure 2:**
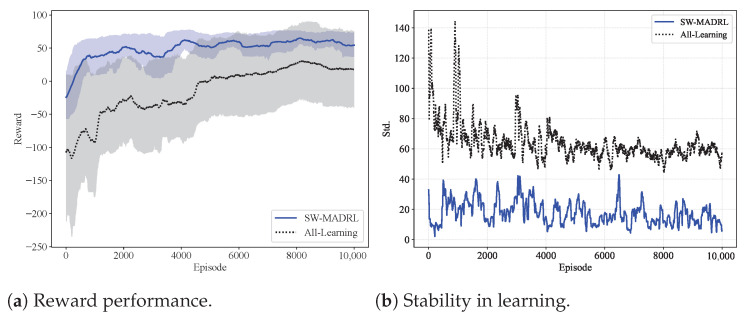
Convergence of SW-MADRL framework.

As shown in Figure 2a, we compare the convergence behavior of the SW-MADRL framework. To highlight the training efficiency improved by the strong-weak learning design, we also include a scheme where all UAVs are trained using MADRL method (denoted as the All-Learning method). We observe that SW-MADRL achieves better reward learning performance. This is because SW-MADRL involves fewer agents, which reduces the complexity of multi-agent interactions thus improving the overall learning efficiency. The shaded areas represent the fluctuations during training. We observe that the SW-MADRL method exhibits smaller fluctuations compared with the All-Learning method. The reduced number of agents in SW-MADRL weakens mutual interference, making the multi-agent learning process easier. This observation is further supported by the variance results shown in Figure 2b, where SW-MADRL demonstrates significantly higher stability than the All-Learning method.

**Figure 3:**
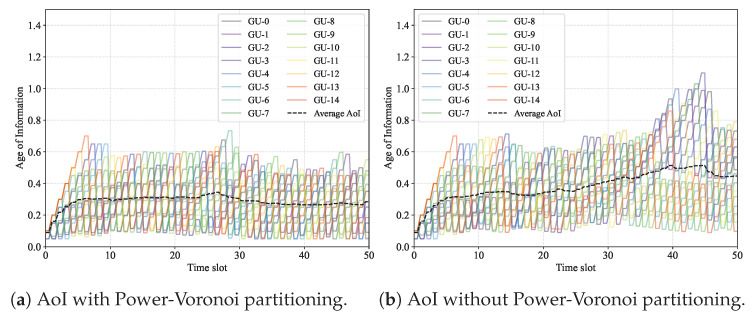
AoI performance improved by Power-Voronoi partitioning.

Figure 3 illustrates the AoI dynamics of all GUs during the transmission process. We design a comparison scheme that does not apply the Power-Voronoi partitioning, denoted as the No-Voronoi method. In Figure 3a, we show the AoI dynamics achieved with the Power-Voronoi partitioning. We observe that the average AoI of the GUs remains stable at around 0.35. However, as shown in Figure 3b, the AoI in the No-Voronoi case gradually exceeds 0.4 in the later stage. This is because the Power-Voronoi partitioning method adapts the H-UAV–L-UAV association based on the real-time traffic states, which balances the traffic load among the H-UAVs and thus improves the overall transmission efficiency. In contrast, the No-Voronoi method lacks traffic-balancing capability, leading to more contention during data transmissions and resulting in poorer AoI performance for the GUs.

### 5.2. Trajectory Planning of the SW-MADRL Framework

**Figure 4:**
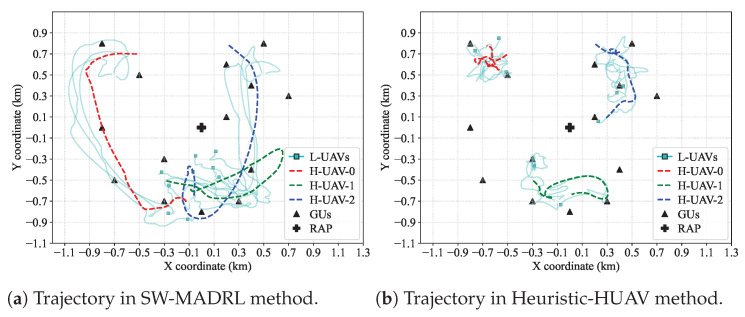
Trajectories under different methods.

We evaluate the UAV trajectory planning results under different methods in Figure 4. We consider the Heuristic-HUAV method as a comparison, where each H-UAV moves heuristically toward the geometric center of its nearby L-UAVs. Figure 4a shows the UAV trajectories generated by the proposed SW-MADRL method. We observe that the L-UAVs, guided by the H-UAVs, are able to cover the entire area and efficiently collect data from the GUs. This is because the H-UAVs, controlled by the MADRL method, possess global planning capabilities through their exploration mechanism. Under this guidance, the L-UAVs also benefit from global planning, enabling coordinated coverage of the entire area. By adopting the strong–weak agent design, the SW-MADRL framework strikes a balance between trajectory planning performance and computational complexity. Consequently, the system requires only limited computational resources while still ensuring comprehensive service coverage for the GUs. However, as shown in Figure 4b, the L-UAVs only perform data collection within local areas. This is because, in the Heuristic-HUAV method, both the H-UAVs and L-UAVs are controlled by predefined rules, which limits them to local planning capabilities. Thus, it becomes difficult to efficiently collect data from GUs across the entire area.

**Figure 5:**
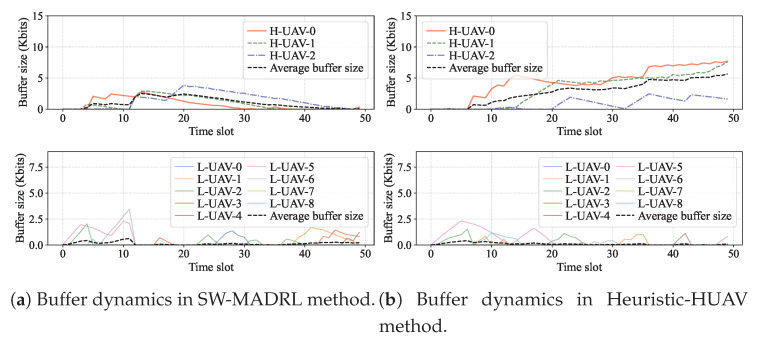
Buffer dynamics under different methods.

To investigate the impact of different trajectory strategies, we illustrate the buffer dynamics of the H-UAVs and L-UAVs during data collection, as shown in Figure 5. Figure 5a shows the buffer dynamics under the SW-MADRL method. We observe that the average buffer size of the L-UAVs remains stable within 1 Kbits, and the average buffer size of the H-UAVs stays within 4 Kbits. However, under the Heuristic-HUAV method, the average buffer size of the L-UAVs also remains relatively low, but the buffer size of the H-UAVs continues to increase over time, as shown in Figure 5b. This is because although the H-UAVs in the Heuristic-HUAV method can efficiently collect data from the L-UAVs, they struggle to maintain a good connection to the RAP. As such, the transmission becomes inefficient, causing data to accumulate in the H-UAV buffers.

### 5.3. AoI Performance Under Different Methods

**Figure 6:**
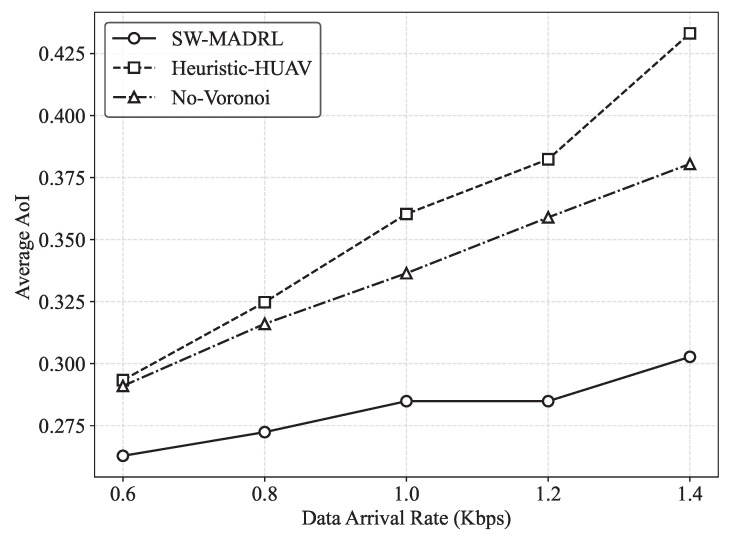
AoI performance under different GU data arrival rates.

In Figure 6, we study the AoI performance of different methods under various GU data arrival rates. The GU data arrival rate is evaluated from 0.6 Kbps to 1.4 Kbps. We observe that as the arrival rate increases, the proposed SW-MADRL method consistently achieves the best AoI performance. Given the GUs’ data arrival rate of 1.4 Kbps, SW-MADRL reduces the AOI by 30% and 21%, compared to No-Voronoi and Heuristic-HUAV, respectively. This is because SW-MADRL not only enables global data collection but also dynamically adjusts the H-UAV–L-UAV association according to the current traffic conditions. This allows the UAVs to coordinate more efficiently, which reduces transmission contention and improves overall transmission efficiency. However, the No-Voronoi method cannot effectively adjust the L-UAV–H-UAV association, which increases contention and consequently reduces the transmission efficiency from the L-UAVs to the H-UAVs. Meanwhile, the Heuristic-HUAV method fails to ensure efficient transmission from the H-UAVs to the RAP, which also results in degraded AoI performance.

**Figure 7:**
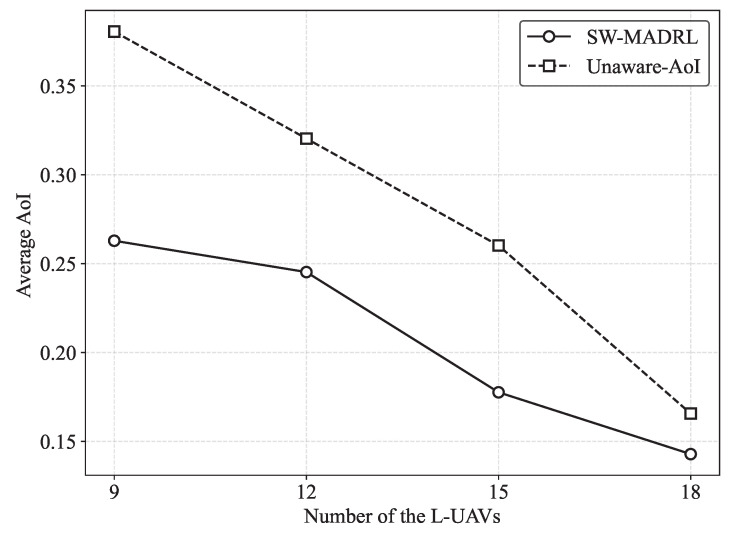
AoI performance under different numbers of L-UAVs.

To further evaluate scalability, we investigate the performance of the proposed SW-MADRL framework under different numbers of L-UAVs. We also introduce an additional benchmark referred to as the Unaware-AoI scheme, in which the AoI term is excluded from the reward design. As shown in Figure 7, the average AoI decreases as the number of L-UAVs increases, since more L-UAVs enhance the service coverage of GUs and thereby improve data collection efficiency. When the number of L-UAVs is relatively small, the proposed SW-MADRL achieves significantly lower AoI compared with Unaware-AoI. This gain is attributed to the AoI-aware reward design, which enables H-UAVs to more effectively coordinate and guide the L-UAVs to reduce the average AoI of the system. As the number of L-UAVs further increases, the performance gap between the two schemes gradually narrows, because the substantially improved coverage allows timely information collection even without explicit AoI awareness. These results demonstrate that the proposed SW-MADRL framework can maintain superior performance, particularly in scenarios with limited L-UAV numbers.

## 6. Discussion

In this section, we analyze the specific advantages of the proposed hierarchical framework compared to existing paradigms and critically address the system’s limitations and potential implementation challenges.

### 6.1. Comparison with Existing Paradigms

The numerical results demonstrate that the proposed SW-MADRL framework significantly outperforms baseline methods in terms of Age of Information (AoI) reduction and convergence stability. This superior performance stems from the architectural decision to decouple global coordination from local execution, addressing distinct deficiencies found in current state-of-the-art approaches.

First, in contrast to centralized optimization methods that formulate data collection, our approach offers superior adaptability. While optimization methods provide theoretically optimal solutions for static snapshots, they suffer from NP-hard computational complexity and fail to adapt to real-time dynamic GU activation. Our results confirm that while heuristic baselines—often used as proxies for rigid planning—struggle with dynamic loads (see Figure 6), our learning-based approach maintains low AoI by adapting partition weights $w_{k}\left(t\right)$ in real-time.

Second, our framework offers a significant improvement over pure MARL. A key finding in our convergence analysis (Figure 2) is that the Strong-Weak agent design converges faster and more stably than the All-Learning baseline. In standard MARL approaches like QMIX or MADDPG, treating every UAV as a learning agent leads to a non-stationary environment where the joint state-action space expands exponentially with swarm size. By limiting learning to the sparse layer of H-UAVs and relegating L-UAVs to reactive Vicsek rules, we effectively reduce the dimensionality of the learning problem, solving the scalability-coordination dilemma.

Finally, the proposed method enhances bio-inspired swarm algorithms. While pure swarm intelligence (e.g., standard Vicsek or PSO) ensures collision avoidance and cohesion, it inherently lacks a global objective function. Our Weighted Vicsek model bridges this gap by injecting goal-oriented vectors, specifically AoI attraction and Link Quality, into the alignment rules. The trajectory analysis (Figure 4) clearly shows that without the strategic shepherding provided by the H-UAVs’ dynamic partitioning, L-UAVs fail to achieve uniform coverage, clustering instead of exploring, which degrades system-wide freshness.

### 6.2. Advantages of the Hierarchical Architecture

The primary advantage of the proposed system is its scalability. The computational load on the H-UAVs grows with the number of other H-UAVs (for Voronoi partitioning) but remains relatively independent of the number of L-UAVs, as L-UAV control is fully decentralized. This decoupling allows the swarm size to increase without overwhelming the central learners. Secondly, the system exhibits robustness to heterogeneity. By explicitly designing for two tiers of capabilities, we avoid the bottleneck of requiring high-performance processors on all nodes. The Power-Voronoi partitioning acts as a flexible load-balancing mechanism. As shown in Figure 5, this adaptability prevents specific H-UAVs from becoming data bottlenecks, which is a common failure mode in static clustering approaches.

### 6.3. Limitations and Challenges

Despite the promising results, several limitations and challenges remain for practical deployment. A significant challenge lies in communication overhead and latency. Our simulation assumes perfect synchronization between sub-slots $\tau_{1}$ and $\tau_{2}$. In practice, the exchange of state information (queue lengths, partition weights) between H-UAVs and the dissemination of these parameters to L-UAVs incurs control overhead. If the channel coherence time is shorter than the control loop latency, the channel-aware weights in the Vicsek model may become outdated, potentially degrading performance.

Furthermore, the system relies on effective NOMA, assuming standard SIC. In low-cost L-UAV deployments, hardware impairments such as phase noise and carrier frequency offset can lead to residual self-interference and imperfect SIC, potentially lowering the achievable data rates. Finally, regarding energy constraints, while we modeled transmit power, we did not incorporate a detailed propulsion energy consumption model for the rotary-wing UAVs. Future work must integrate energy-aware constraints directly into the Vicsek force vectors to extend the swarm’s operational lifetime.

## 7. Conclusions

This paper proposed a hierarchical data collection framework for heterogeneous UAV-assisted WSNs, integrating MADDPG-based global coordination with self-organized local swarming. By employing dynamic power-Voronoi partitioning and a weighted Vicsek model, the proposed SW-MADDPG scheme effectively balances global workload distribution with local adaptability under incomplete environmental information. Experiments demonstrate that our approach ensures scalability and reduces the Age of Information (AoI) by 30% and 21% compared to static partitioning and heuristic baselines, respectively. This spatial-temporal decomposition offers a practical solution for large-scale sensing tasks where centralized control is computationally prohibitive. Future work will focus on enhancing the framework’s robustness against communication constraints, supporting heterogeneous user requirements, and validating the system on physical UAV platforms.

## Figures and Tables

**Table 1:** Summary of Key Notations and Symbols.

Symbol	Description
**System Parameters and Sets**	
$N_{H}$, $N_{L}$, $N_{G}$	Set of H-UAVs, L-UAVs, and GUs, respectively
Δt	Duration of one time slot
$\tau_{1},\tau_{2}$	Duration of sub-slots 1 and 2, where $\tau_{1}+\tau_{2}=\Delta t$
**Channel Model and Communication**	
$h_{ab}\left(t\right)$, $d_{ab}\left(t\right)$	Channel gain and distance between nodes *a* and *b* at time *t*
$\sigma^{2}$	Power of additive white Gaussian noise (AWGN)
$P_{i}^{g},P_{j}^{l},P_{k}^{h}$	Transmit power of GU *i*, L-UAV *j*, and H-UAV *k*
**UAV Dynamics and Constraints**	
$u_{k}\left(t\right),v_{k}\left(t\right)$	Position and velocity of H-UAV *k* at time *t*
$p_{l}\left(t\right),v_{l}\left(t\right)$	Position and velocity of L-UAV *l* at time *t*
$v_{\max}^{H},a_{\max}^{H}$	Maximum velocity and acceleration of H-UAVs
$d_{\min}^{HH},d_{\min}^{LL}$	Minimum separation distance between H-UAVs and L-UAVs
**Power-Voronoi Partitioning**	
$V_{k}\left(t\right)$	Power-Voronoi cell associated with H-UAV *k* at time *t*
$w_{k}\left(t\right)$	Adaptive weight for H-UAV *k* in power-Voronoi diagram
$L_{k}^{g}$, $L_{k}^{l}$	Set of GUs and L-UAVs within cell $V_{k}\left(t\right)$
$\Lambda_{k}\left(t\right)$	Total data generation rate within cell $V_{k}\left(t\right)$
$U_{k}\left(t\right)$	Area of power-Voronoi cell $V_{k}\left(t\right)$
**SINR and Data Rates**	
$\gamma_{ij}^{\left(1\right)}\left(t\right)$	SINR for GU *i* to L-UAV *j* link in sub-slot $\tau_{1}$
$\gamma_{jk}^{\left(2\right)}\left(t\right)$	SINR for L-UAV *j* to H-UAV *k* link in sub-slot $\tau_{2}$
$\gamma_{k0}\left(t\right)$	SINR for H-UAV *k* to RAP link
$R_{ij}^{\left(1\right)}\left(t\right)$	Data rate from GU *i* to L-UAV *j* in sub-slot $\tau_{1}$
$R_{j}^{\left(2\right)}\left(t\right)$	Data rate from L-UAV *j* to H-UAV *k* in sub-slot $\tau_{2}$
$R_{k0}\left(t\right)$	Data rate from H-UAV *k* to RAP
$R_{k}^{\mathrm{NOMA}}\left(t\right)$	Sum-throughput from all L-UAVs to H-UAV *k*
**Queue Dynamics and Scheduling**	
$Q_{j}^{\left(L\right)}\left(t\right)$, $Q_{k}^{\left(H\right)}\left(t\right)$	Data queue length at L-UAV *j* and H-UAV *k* at time *t*
$Q_{\max}$	Maximum buffer capacity
$\beta_{ij}\left(t\right)$	Scheduling variable indicating GU *i* transmits to L-UAV *j*
$B_{i}\left(t\right)$	Packet size of GU *i* at time *t*
$\lambda_{i}$	Data generation rate of GU *i*
**Age of Information (AoI)**	
$\Delta_{i}\left(t\right)$	Age of Information of GU *i* at time *t*
$D_{i}\left(t\right)$	End-to-end latency of GU *i*’s packet
$g_{i}\left(t\right)$	Indicator whether GU *i*’s packet was generated in slot *t*
$s_{i}\left(t\right)$	End-to-end success indicator for GU *i*
$\Delta_{\min},\Delta_{\max}$	Minimum and maximum AoI for normalization
**Weighted Vicsek Model**	
$u_{l}\left(t\right)$	Unnormalized direction vector for L-UAV *l*
$w_{lj}$	Alignment weight between L-UAVs *l* and *j*
$f_{l}^{\mathrm{AoI}}\left(t\right)$	AoI-weighted attraction force
$f_{l}^{\mathrm{link}}\left(t\right)$	Link quality enhancement force
$f_{l}^{\mathrm{cong}}\left(t\right)$	Congestion avoidance force
$f_{l}^{\mathrm{bdry}}\left(t\right)$	Boundary confinement force
$\omega_{i}^{\mathrm{AoI}}\left(t\right)$	AoI weight for GU *i*
$\omega_{l}^{\mathrm{link}}\left(t\right)$	Link quality weight for L-UAV *l*
$\alpha_{\mathrm{AoI}},\alpha_{\mathrm{link}},\alpha_{\mathrm{cong}},\alpha_{\mathrm{bdry}}$	Weight parameters for different forces
$r_{s},r_{\min},r_{b}$	Sensing radius, minimum separation radius, boundary threshold
**MADRL Framework**	
S	Global state space
$O_{k}$, $A_{k}$	Local observation space and action space of H-UAV *k*
$o_{k}\left(t\right)$, $a_{k}\left(t\right)$, $r_{k}\left(t\right)$	Local observation, action, and local reward of H-UAV *k* at time *t*
$\mu_{k}\left(\cdot \right),Q_{k}\left(\cdot \right)$	Actor and critic networks for H-UAV *k*
$\theta_{k},\phi_{k}$	Parameters of actor and critic networks
$\gamma_{rl}$	Discount factor for reinforcement learning

## Data Availability

The data presented in this study are available on request from the corresponding author due to the data being part of an ongoing study.
